# Double systemic cytokine release syndrome following sequential infusion of anti-CD22 and anti-CD19 chimeric antigen receptor T cells after autologous hematopoietic stem cell transplantation for a central diffuse large B-cell lymphoma patient: A case report and literature review

**DOI:** 10.3389/fimmu.2023.1098815

**Published:** 2023-01-31

**Authors:** Jing Zheng, Yao Xiao, Xue Q. Wu, Qiong Z. Xiao, Chun Feng, Kai B. Gao

**Affiliations:** ^1^ Departments of Hematology, The First People s’ Hospital of Yichang, China Three Gorges University, Yichang, Hubei, China; ^2^ Departments of Internal Medicine, People’s Hospital of Wufeng Tujia Autonomous County, Yichang, Hubei, China

**Keywords:** chimeric antigen receptor T cell, cytokine release syndrome, central diffuse large B-cell lymphoma, immunotherapy, adverse reactions

## Abstract

**Background:**

Chimeric Antigen Receptor T cell(CAR T-cell) therapy has been a great success in relapsed/refractory acute B lymphoblastic leukemia and B-cell lymphoma. At the same time, there are also related adverse reactions, especially cytokine release syndrome(CRS) and immune effector cell associated neurotoxicity syndrome(ICANS). However, Double CRS caused by CRA T cells are very rare.

**Case report:**

Here, we report a 33-year-male with secondary central diffuse large B-cell lymphoma(CNSL) who develpoed double CRS following sequential infusion of Anti-CD22 and Anti-CD19 CAR T cells after autologous hematopoietic stem cell transplantation(ASCT). On d+5, the patient developed high fever, along with chilly sensation, shivering, headache, blood oxygen desaturation, shock, weakness, severe thirst, and heart rate decline. IL-6 and ferritin increased significantly. The patient was diagnosed with the first CRS (grade 3). On d+36, the patient again had a persistent fever(T>39C) and limbs rash. IL-6 and ferritin again increased significantly on d+38. After exclusion of infection, a diagnosis of double CRS was made. The patient’s symptoms were completely relieved after receiving tocilizumab, glucocorticoids, and other supportive treatments on d+45.On d+90, contrast-enhanced MR angiogram shows that the lesion basically disappeared, indicating the patient had achieved CR. At the end of the follow-up at d+150, the patient was functioning normally without any sequelae.

**Conclusion:**

This is the first reported case worldwide where the patient with secondary CNSL suffered double CRS after CAR T-cell infusion. Our findings showed that it is important to increase awareness of early detection and diagnosis of double CRS and adopt appropriate treatment strategies.

## 1 Introduction

In the recent 10 years, chimeric antigen receptor T cell (CAR T-cell) therapy has made a great breakthrough in the treatment of B-cell malignancies. The most typical example is that CD19-CAR T cell treatment significantly increases the response rate and prolongs the survival of relapsed/refractory acute lymphoblastic leukemia and B-cell lymphoma (1, 2). Central nervous system lymphoma (CNSL) is a rare and fatal subtype of non-Hodgkin’s lymphoma that is rarely treated with CAR T cell therapy due to severe CART-related complications. However, Wu reported 13 CNSL patients (four, primary CNSL; nine, secondary CNSL) with autologous stem cell transplantation (ASCT) sequential CD19/22 CAR T-cell infusion (3). The overall response (OR) and complete remission (CR) rates were 81.81% and 54.55%, respectively. The 1-year overall survival rate (OS) was 82.5%. In addition, only 7.7% of CNSL patients developed severe immune effector cell-associated neurotoxicity syndrome (ICANS) and no patient suffered grades 3-4 cytokine release syndrome (CRS). In conclusion, CAR T-cell therapy is a safe and effective treatment for CNSL. However, double CRS caused by CRA T cells is very rare. Herein, we report a case of a patient with secondary CNSL who developed double CRS after ASCT combined with anti-CD22 and anti-CD19 CAR-T cell therapy.

## 2 Case presentation

In August 2021, a 33-year-old man presented with a left neck mass for 1 month and was admitted to the Department of Hematology in our hospital for examination. Physical examination indicated multiple enlarged lymph nodes in the neck and axilla. Contrast-enhanced computed tomography (CT) examination showed multiple enlarged lymph nodes in the neck, submandibular region, upper and lower clavicular fossae, axilla, mediastinum, intraperitoneal and retroperitoneal regions, and groin. Cervical lymph node pathology confirmed a diagnosis of diffuse large B-cell lymphoma (DLBCL). Immunohistochemical analysis showed the following results for lymphoma cells: CD3 (-), CD10 (-), CD20 (+), BCL2 (+,60%), BCL6 (+,80%), CD43 (+), C-MYC (-), CyclinD1 (-), CD21 (-), and Ki-67 (+, 70%). EBER by *in situ* hybridization test was negative in the lymphoma cells. The fluorescence *in situ* hybridization test yielded positive results for BCL-6 rearrangement, but negative results for MYC and BCL-2 rearrangement. Next-generation sequencing of lymphoma cells revealed a CD58 mutation (exon 3, 5.4%) coinciding with CREBBP (exon 2, 12%), FAS (exon 9, 9.9%), KMT2C (exon 36, 11.4%), SOCS1 (exon 2, 7.5%), and TNFRSF14 (exon 1, 7.2%) mutations. Therefore, this patient was diagnosed with DLBCL stage IVA, International Prognostic Index score of 3, and was considered at high-intermediate risk.

The patient received six cycles of rituximab, cyclophosphamide, adriamycin, vincristine, and prednisone chemotherapy (R-CHOP), and two cycles of rituximab. After chemotherapy, the efficacy was evaluated as CR by positron emission tomography-CT (PET-CT).

**Figure 1:**
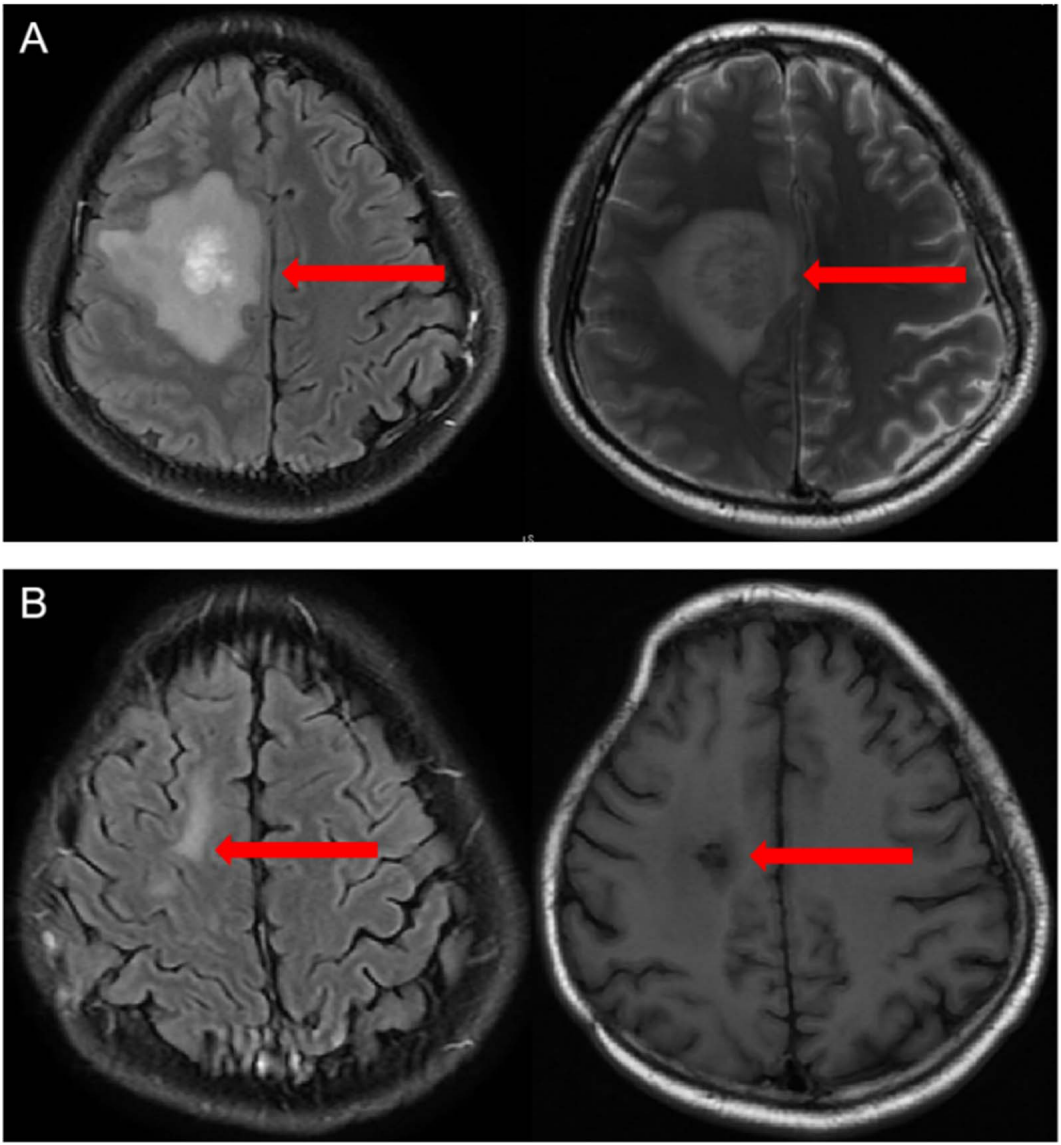
**(A)** Contrast-enhanced magnetic resonance angiogram showing areas of signal abnormality in both the right temporal lobe and frontal lobe with areas of contrast enhancement in March 2022 **(B)** Contrast-enhanced magnetic resonance angiogram showing that the lesion disappeared without obvious enhancement 3 months after the CAR T cell infusion, indicating that the patient achieved complete remission. The red arrow represents the lesions.

In March 2022, the patient developed left limb weakness and headache. Brain enhanced magnetic resonance imaging showed lymphoma in the right temporal lobe and frontal lobe, accompanied by peripheral cerebral edema and right subfalcine herniation (**Figure 1**). He underwent emergency decompressive craniectomy and tumor biopsy due to worsening symptoms of cerebral herniation. The diagnosis of DLBCL was confirmed by pathology, which demonstrated CD19 (50%+), CD22 (80%+), CD20 (80%+), Bcl-6 (60%+), Mum- 1(+), c-myc(40%+), and Ki-67 (60%+). Next generation sequencing of lymphoma cells revealed TP53 mutation coinciding with

MYD88 (exon5, 20.99%), CD79B (exon4, 1.81%), CREBBP (exon2, 15.81%), KMT2C (exon36, 6.86%), and KMT2D (exon31, 14.66%) mutations. Then, the patient received three cycles of rituximab, methotrexate, cytarabine, and obrutinib chemotherapy. After chemotherapy, the efficacy was evaluated as a partial response (PR) by PET-CT.

## 3 CD22/CD19 CAR T-cell therapy following Autologous stem cell transplantation

Since previous reports showed that combining CAR- 19/22 T cell therapy with ASCT had a curative effect in the treatment of refractory and recurrent DLBCL with TP53 alterations (especially central nervous system lymphoma) (3, 4), the patient was enrolled in the clinical trial with the approval of the ethics committee of the First People’s Hospital of Yichang, China Three Gorges University (PJ-JS2022-02) and with the patient’s informed consent.

**Figure 2:**
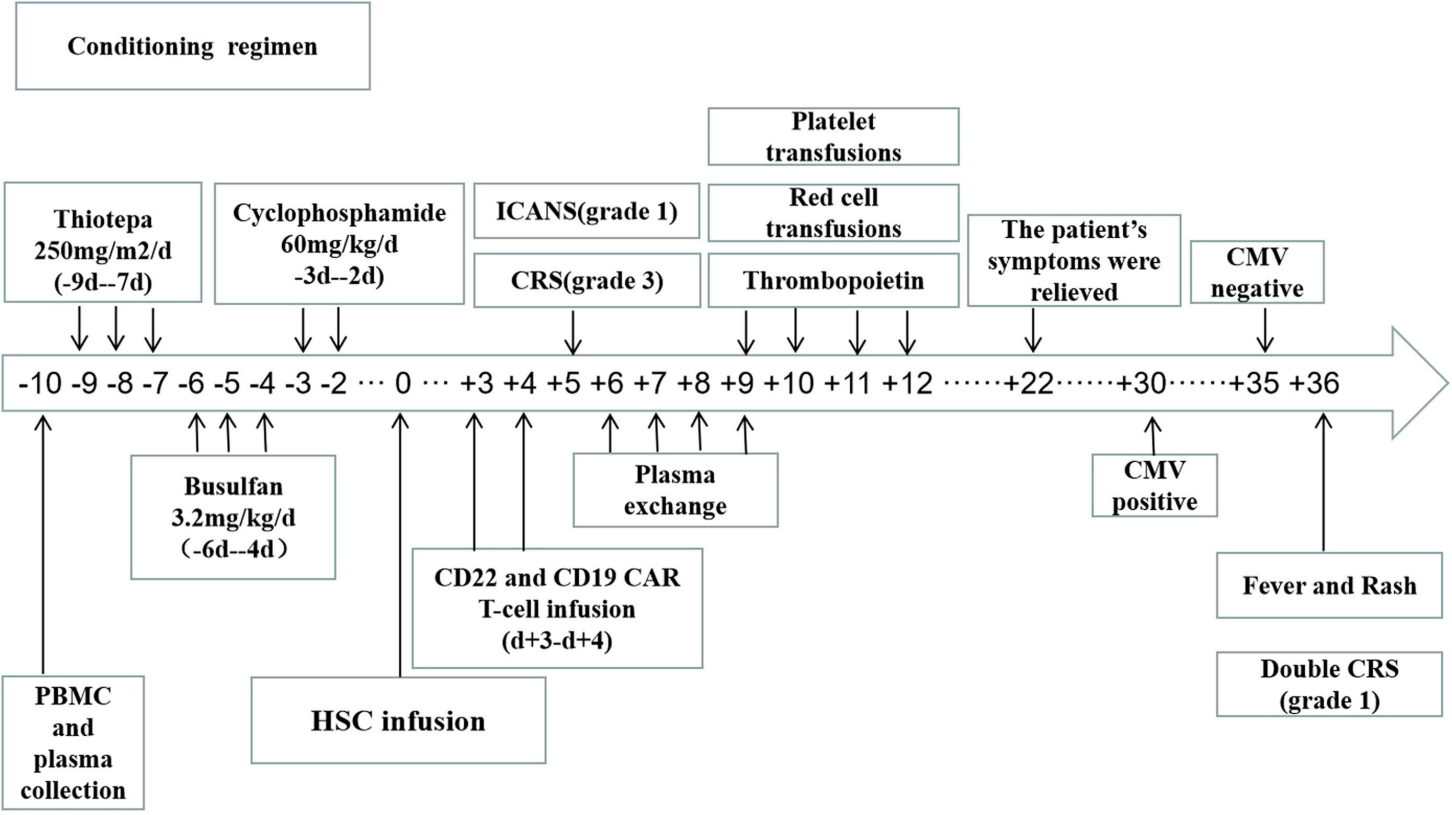
The patient underwent peripheral blood mononuclear cell and plasma collection on d-10 and received conditioning regimen before HSC infusion on do. Two separate CAR T-cell products (CD22 and CD19 CAR T-cells) were infused within the range of 3-4 days (d+3 to d+4) after HSC infusion.

The conditioning regimen was mainly a thiotepa-based protocol, which included thiotepa 250 mg/(m2·d) from days -9 to -7, busulfan 3.2 mg/(kg ·d) from days -6 to -4, and cyclophosphamide 60 mg/(kg ·d) from days −3 to −2. Autologous stem cells (13 × 106/Kg CD34+ stem cells) were transfused on d0. Then, two CAR T-cell products (2×106/Kg CD22 CAR T cells and 2×106/Kg CD19 CAR T cells) were infused in the patient for 2 consecutive days (d+3 to d+4), respectively (**Figure 2**). The two CAR T cells were provided by Shanghai Yake Co. Ltd (Shanghai, China). A lentiviral vector was used to carry a second generation CD19-directed CAR from a murine antibody phage display library with a 4-1BB co-stimulatory and CD3ζ signaling domains. In addition, The antigen recognition domain of this CD22 specific CAR was gained from a human antibody phage display library. A lentiviral vector carrying two CD19/22 CAR with 4-1BB costimulatory domain and a CD3-zeta signaling domain was constructed as previously described (5).

## 4 Related toxicity management of CD22/CD19 CAR T-cell therapy

### 4.1 CRS and ICANS

**Figure 3:**
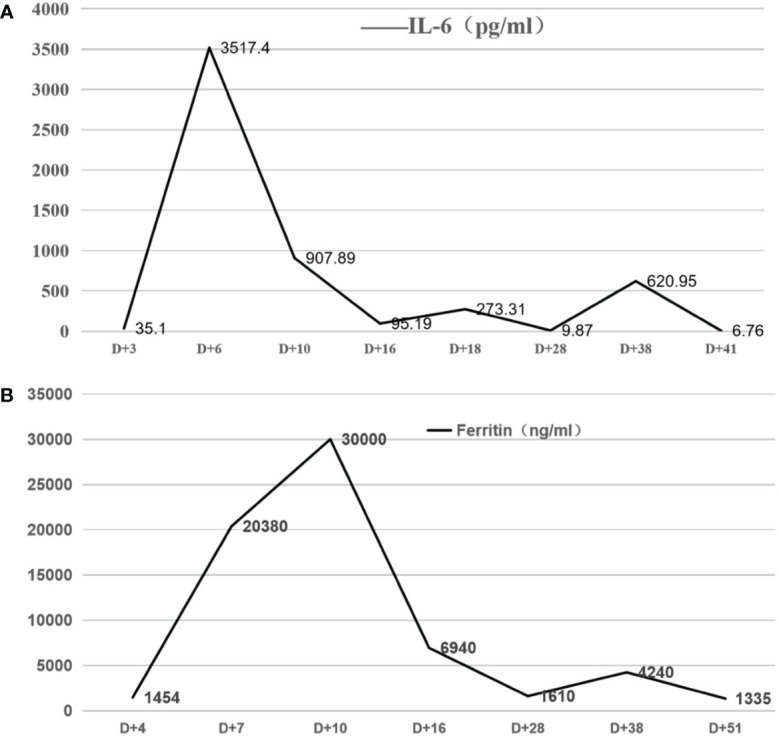
**(A, B)** The serum IL-6 and ferritin levels at different time points after CAR-T cell infusion After CAR T-cell infusion, the patient developed fever and elevation of IL-6 and ferritin levels on d+5. The IL-6 level peaked for the first time on d+6, while the ferritin level peaked for the first time on d+10. The levels of IL-6 and ferritin significantly increased again on d+38.

On d+5, the patient developed a high fever, with the highest temperature being 40°C, along with a chilly sensation, shivering, headache, blood oxygen desaturation, shock, weakness, severe thirst, and heart rate decline, as low as 30 beats per minute. The patient was diagnosed with CRS (grade 3) and ICANS (grade 1). After CAR T cell infusion, IL-6 and ferritin levels increased significantly. The IL-6 level peaked on d+6, while that of ferritin peaked on d+10 (**Figure 3**). Therefore, the patient received tocilizumab, dexamethasone, intravenous immunoglobulin, oxygen, vasoactive agents, isoproterenol, and plasma exchange. The patient’s symptoms were relieved on d+22. Meanwhile, the IL-6 and ferritin levels almost normalized. This patient was infected with cytomegalovirus on d+30 with a cytomegalovirus level of up to 900copies/ml. The CMV DNA turned negative after antiviral therapy with ganciclovir on d+35. On d+36, the patient again had a persistent fever (T>39C) and a limb rash. IL-6 and ferritin again increased significantly on d+38(**Figure 3**). Some laboratory tests, including CRP, PCT, and metagenomic next-generation sequencing, were normal. In addition, the patient continued to have intermittent fevers while receiving broad-spectrum antibiotics. After the exclusion of infection, a diagnosis of double CRS was made. The patient was treated with glucocorticoids. The above symptoms and inflammatory indicators normalized on d+45.

### 4.2 Simulated hemophagocytic lymphohistiocytosis

On the 9th day after CD19 CAR T-cell infusion, the patient had a persistent fever, hyperlipemia, and a progressive decrease in the levels of hemoglobin and platelets. Hemophagocytosis was observed in the bone marrow. At the same time, the levels of fibrinogen, ferritin, and sIL-2R were <1.5 g/L, >30000 mg/L, and >7500 U/ml, respectively. HLH was diagnosed according to the HLH 2004 criteria. The patient was treated with tocilizumab, dexamethasone, intravenous immunoglobulin, and plasma exchange. On d+27, these indicators had almost normalized.

### 4.3 Blood routine and coagulation function

From d-3 after receiving pretreatment, the patient gradually developed neutropenia, thrombocytopenia, and anemia. We treated him with infusion of red blood cells, platelets, and human thrombopoietin. We did not use G-CSF because it might aggravate the degree of CRS. With autologous stem cell infusion, the absolute neutrophil count was 2.1×10^9^/L on D+10 and platelet count was 59×10^9^/L on D+7, indicating granulocytes and megakaryocytes were transplanted. In view of CAR T cell expansion on d+11, the patient again experienced a progressive decrease in the levels of hemoglobin and platelets. In addition, the patient also developed coagulation function changes, mainly hypofibrinemia.These changes were transient and eventually normalized after symptomatic treatment.

### 4.4 CAR T cell kinetics

**Figure 4:**
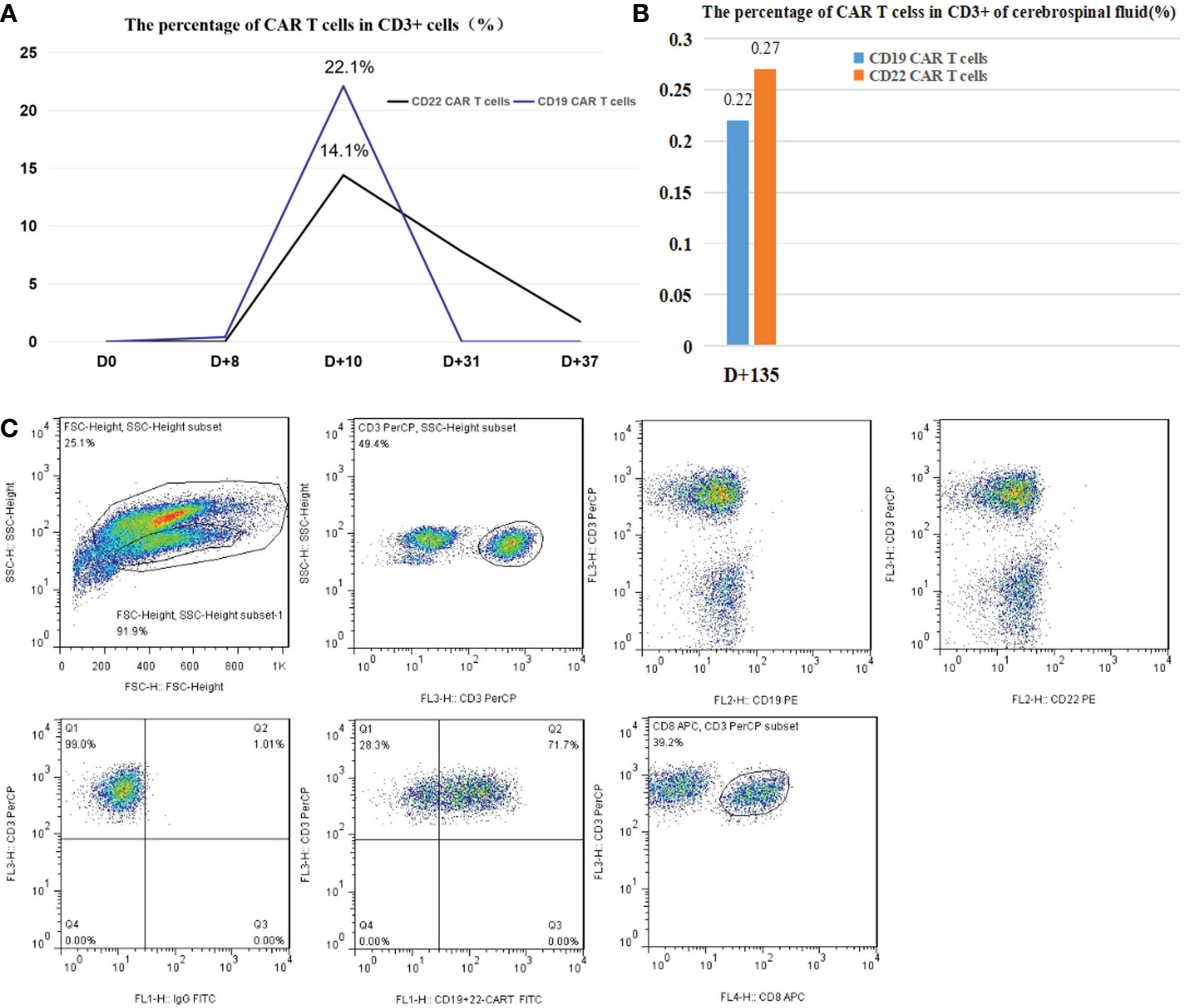
**(A)** The peak percentages of CAR T cells in CD3+ cells in peripheral blood were 22.1% and 14.4%, as shown. **(B)** On d+135, the percentages of CD19 and CD22 CAR T cells in CD3+ cells of the cerebrospinal fluid were 0.22% and 0.27%, respectively. **(C)** Representative plots of the flow cytometry gating strategy for anti-CD19 and anti-CD22 CAR T-cell identification. The ratio of CAR T cells to CD3+ T cells was determined by flow cytometry using a proprietary anti-CD19 and anti-CD22 CAR-T cell-specific detection reagent(Shanghai YaKe), and the monoclonal antibodies and matching reagents, such as CD3-PerCP BD SK7, CD8-APC BD SKI. CD19-PE BD 4G7, CD22-PE BD S-HCL-1, and IgG-FITC BD X40, were obtained from BD.

After the first infusion of CD22/CD19 CAR T cells, the CD19 and CD22 CAR T cells in the peripheral blood reached the expansion peak on d+10. However, CD19 CAR T cells in the peripheral blood were almost undetectable on d+31 (**Figure 4**). CAR T cell testing in cerebrospinal fluid was not performed during the double CRS because the patient refused to undergo a lumbar puncture. To remedy this regret, the patient underwent lumbar puncture on d+135. Fortunately, the percentages of CD19 and CD22 CAR T cells in CD3+ cells of the cerebrospinal fluid (CSF) were 0.22% and 0.27%, respectively.

### 4.5 Therapeutic evaluation and follow-up

On d+90, contrast-enhanced MR angiography showed that the lesion had disappeared without obvious enhancement, indicating that the patient had achieved CR (**Figure 1**).

## 5 Discussion

Recently, CAR T-cell therapy has been a great success in relapsed/refractory B-cell malignancies. However, treatment-related complications, especially CRS, restrict the development and application of CAR T-cell therapy. CRS is a common immune-related toxicity associated with CAR T-cell therapy and can be life-threatening in some patients, while double CRS is very rare. We first reported the occurrence of double CRS following sequential infusion of anti-CD22 and anti-CD19 CAR T-cells after ASCT for a secondary CNSL patient.

It has been reported in the literature that CRS was observed in 37% to 93% of patients with hematologic malignancies treated with autologous CD19-CAR T cells (6–8). Wang T analyzed the safety of 21 patients with DLBCL treated with ASCT-CART, and the results showed that 71% of patients developed grade 1-2 CRS and 5% of patients suffered from grade 3-4 CRS (9). The incidence and degree of CRS depend on tumor type, tumor burden, CAR-T cell dose, co-stimulatory domain type, CAR T cell activation, and expansion (10). The mechanism of CRS may be related to inflammatory cytokines from immune cells and monocytes/macrophages (11).

With the infusion of CAR-T cells, patients may experience significant serum elevation of multiple cytokines, including IL- 1, IL-2, IL-6, IL- 10, and interferon-gamma (INF- γ) (12). Monocytes/macrophages are mediators of CRS and can be responsible for the release of IL-6, IL- 1, ferritin, and INF- γ, which results in endothelial activation and the secretion of von Willebrand factor (VWF) and angiopoietin-2 from endothelial cells.

Most CRS occur within 1 to 14 days after CAR T-cell infusion (13). Very few late-onset CRS and chronic CRS occur more than 2 weeks after CAR T-cell infusion. Here, we reported the first case of double CRS after anti-CD22 and anti-CD19 CAR

T cell therapy: the first CRS occurred on d+5 and the second CRS emerged on d+36. The serum levels of IL-6 and ferritin significantly increased in both CRS stages, which was consistent with the literature reports (12). The second CRS showed fever, rash, and a marked increase in cytokines, consistent with the diagnosis of CRS after the exclusion of infection. On d+31, CD19-CAR T cells could not be detected and serum CD22-CAR T cells level were significantly reduced. In other words, no CAR T cell expansion in peripheral blood was detected during the second onset of CRS. After treatment with dexamethasone, the symptoms of the second CRS were completely relieved.Even though CART were not detectable at d+35 that local expansion potentially in the CNS where the tumor lesion is was driving the secondary CRS. It is speculated that the mechanism of the second CRS may be related to local CAR T-cell immune response and cytokines. This speculation was confirmed by the detection of CAR T cells in CSF. In addition, the severity and cytokine levels of the first CRS were much higher than those of the second CRS, which was consistent with the activation and expansion levels of CAR T cells.

Common treatment regimens for CRS include tocilizumab (IL-6 receptor-directed antibody), glucocorticoids, anakinra (IL-1 receptor antagonist), plasmapheresis, and other supportive medical care (14). The patient we reported was diagnosed with CRS (grade 3) and was treated with tocilizumab, dexamethasone, intravenous immunoglobulin, oxygen, vasoactive agents, and plasmapheresis. After the above treatment, the symptoms of CRS were completely relieved. Since severe CRS can be life-threatening, appropriate measures should be taken to prevent CRS. First, We should minimize the tumor burden as much as possible before CAR T-cell infusion. Second, the dose of CAR T cells was appropriately reduced without affecting the efficacy. This protocol of CAR T-cell dose fractionation can also be used for CAR T-cell infusion. Third, it is also crucial to select appropriate CAR T-cell products according to different tumor types.

The relationship between CRS and the prognosis of hematological tumors has not been determined yet. Grigor EJM investigated the relationship between CRS and CR in B-cell malignancies by using a scatter plot and found that the incidence and grade of CRS did not correlate with CR (15). More studies are needed to further analyze the relationship between CRS and prognosis such as CR and OS.

Our case report showed the excellent efficacy of sequential infusion autologous anti-CD22 and anti-CD19 CAR T-cell therapy after ASCT for a CNSL. However, CAR T cell-associated toxicity is also very important. To our knowledge, this is the first reported case worldwide where a patient with secondary CNSL suffered double CRS after CAR T-cell infusion. It is important to increase awareness of early detection and diagnosis of double CRS and adopt appropriate treatment strategies. In addition to this, it is necessary to carry out the corresponding basic and clinical studies of CAR T cell-associated double CRS.

## Data availability statement

The raw data supporting the conclusions of this article will be made available by the authors, without undue reservation.

## Ethics statement

The studies involving human participants were reviewed and approved by Ethics Committee of the First People’s Hospital of Yichang. The patients/participants provided their written informed consent to participate in this study. Written informed consent was obtained from the individual(s) for the publication of any potentially identifiable images or data included in this article.

## Author contributions

YX and XW contributed equally as first author to this work. JZ and KG have made significant contribution to data acquisition and manuscript editing. XW and CF has made significant contribution to design and literature review. QX have made significant contribution to data collection and manuscript review. All authors contributed to the article and approved the submitted version.

## References

[1] Zhang LN Song Y Liu D. CD19 CAR-T cell therapy for relapsed/refractory acute lymphoblastic leukemia: Factors affecting toxicities and long-term efficacies. J HeWestin matol Oncol (2018) 11(1):41. doi: 10.1186/s13045-018-0593-5 PMC5855988 29544528

[2] Westin JR Kersten MJ Salles G Abramson JS Schuster SJ Locke FL. Efficacy and safety of CD19-directed CAR-T cell therapies in patients with relapsed/refractory aggressive b-cell lymphomas: Observations from the JULIET, ZUMA- 1, and TRANSCEND trials. Am J Hematol (2021) 96(10):1295–1312. doi: 10.1002/ajh.26301 34310745 PMC9290945

[3] Wu J Meng F Cao Y Zhang Y Zhu X Wang N. Sequential CD19/22 CAR T-cell immunotherapy following autologous stem cell transplantation for central nervous system lymphoma. Blood Cancer J (2021) 11(7):131. doi: 10.1038/s41408-021-00523-2 34267187 PMC8282870

[4] Wei J Xiao M Mao Z Wang N Cao Y Xiao Y. Outcomes of Relapsed/Refractory aggressive b-cell non-Hodgkin lymphoma (r/r b-NHL) patients with TP53 gene disruption treated with CD19/22 cocktail CAR T-cell therapy alone or incorporated with autologous stem cell transplantation (ASCT). Signal Transduct Target Ther (2022) 7(1):101. doi: 10.1038/s41392-022-00924-0 35399106 PMC8995369

[5] Pan J Yang JF Deng BP Zhao XJ Zhang X Lin YH. High efficacy and safety of low-dose CD19-directed CAR-T cell therapy in 51 refractory or relapsed b acute lymphoblastic leukemia patients. Leukemia (2017) 31(12):2587–93. doi: 10.1038/leu.2017.145 28490811

[6] Neelapu SS. Managing the toxicities of CAR T-cell therapy. Hematol Oncol (2019) 37(1):48–52. doi: 10.1002/hon.2595 31187535

[7] Neelapu SS Locke FL Bartlett NL Lekakis LJ Miklos DB Jacobson CA. Axicabtagene ciloleucel CAR T-cell therapy in refractory Large b-cell lymphoma. N Engl J Med (2017) 377(26):2531–44. doi: 10.1056/NEJMoa1707447 PMC5882485 29226797

[8] Lei W Xie M Jiang Q Xu N Li P Liang A. Treatment-related adverse events of chimeric antigen receptor T-cell (CAR T) in clinical trials: A systematic review and meta-analysis. Cancers (Basel) (2021) 13(15):3912. doi: 10.3390/cancers13153912 34359816 PMC8345443

[9] Wang T Xu L Gao L Tang G Chen L Chen J. Chimeric antigen receptor T-cell therapy combined with autologous stem cell transplantation improved progression-free survival of relapsed or refractory diffuse large b-cell lymphoma patients: A single-center, retrospective, cohort study. Hematol Oncol (2022) 40(4):637–44. doi: 10.1002/hon.2975 35141937

[10] Hay KA Hanafi LA Li D Gust J Liles WC Wurfel MM. Kinetics and biomarkers of severe cytokine release syndrome after CD19 chimeric antigen receptormodified T-cell therapy. Blood (2017) 130(21):2295–306. doi: 10.1182/blood-2017-06-793141 PMC5701525 28924019

[11] Hirayama AV Turtle CJ. Toxicities of CD19 CAR-T cell immunotherapy. Am J Hematol (2019) 94(S1):S42–9. doi: 10.1002/ajh.25445 30784102

[12] Maude SL Frey N Shaw PA Aplenc R Barrett DM Bunin NJ. Chimeric antigen receptor T cells for sustained remissions in leukemia. N Engl J Med (2014) 371:1507– 17. doi: 10.1056/NEJMoa1407222 PMC4267531 25317870

[13] Frey N Porter D. Cytokine release syndrome with chimeric antigen receptor T cell therapy. Biol Blood Marrow Transplant (2019) 25(4):e123–7. doi: 10.1016/j.bbmt.2018.12.756 30586620

[14] Sheth VS Gauthier J. Taming the beast: CRS and ICANS after CAR T-cell therapy for ALL. Bone Marrow Transplant (2021) 56(3):552–66. doi: 10.1038/s41409-020-01134-4 PMC8592274 33230186

[15] Grigor EJM Fergusson D Kekre N Montroy J Atkins H Seftel MD. Risks and benefits of chimeric antigen receptor T-cell (CAR-T) therapy in cancer: A systematic review and meta-analysis. Transfus Med Rev (2019) 33(2):98–110. doi: 10.1016/j.tmrv.2019.01.005 30948292

